# Editorial: Pain, immunity, and neurological and autoimmune disorders

**DOI:** 10.3389/fimmu.2023.1195204

**Published:** 2023-04-04

**Authors:** Ping-Heng Tan, Yong-Jing Gao, Yuanpu Peter Di, Jen-Kun Cheng

**Affiliations:** ^1^ Department of Anesthesiology, Chi Mei Medical Center, Tainan, Taiwan; ^2^ School of Medicine, College of Medicine, National Sun Yat-sen University, Kaohsiung, Taiwan; ^3^ Institute of Pain Medicine and Special Environmental Medicine, Nantong University, Nantong, Jiangsu, China; ^4^ Departmental of Environmental and Occupational Health, School of Public Health, University of Pittsburgh, Pittsburgh, PA, United States; ^5^ Department of Anesthesiology, Mackay Memorial Hospital, Taipei, Taiwan

**Keywords:** pain, nociceptor, immune cells, autoimmune disease, inflammation, analgesia

Emerging research shows that nociceptor neurons could significantly affect the immune response and inflammation. Thus, pain is not only a cardinal feature of inflammation but also plays an essential role in regulating immunity. Upon noxious stimulation, nociceptors can release neuropeptides and neurotransmitters that have potent regulatory effects on the vasculature and innate and adaptive immunity. Many immune cells, such as macrophages, mast cells, neutrophils, dendritic cells and T cells, express receptors for these neuronal mediators, allowing them to respond directly to nociceptor activation. Recent reports also revealed that nociceptors play a vital role in regulating joint, gastrointestine, and skin disorders, and targeting pain could also lessen inflammation. Nociceptor neurons also are important in regulating inflammation in gastrointestinal diseases. Dysregulation of these interactions could underlie the pathogenesis of inflammatory diseases in the joints, gastrointestinal tracts and skin. This special issue presents a collection of papers with the associated interaction of nociceptors, immune cells, vagus nerve, rheumatoid arthritis, trigeminal neuralgia, and other types of pain.

Biological interactions between immune cells and neurons are bidirectional, in which immune cells modulate neuronal functions and neurons control immune responses. Nociceptor neurons and the immune system have many parallels and interactions, which Hanč et al. investigated. There is a parallel mechanism and function between the evolution of the sensory and immune systems, which the authors discussed first. The authors identify molecular mechanisms of the interaction and categorize each myeloid cell type individually. Several receptors on immune cells respond to neuron-derived ligands. Finally, they show that the immune system is coordinated by different branches of the peripheral nervous system, including sensory and autonomic neurons. This paper includes valuable information for researchers interested in knowing in-depth about the known effects of nociceptors on the different types of immune cells.

Autism spectrum disorder (ASD) is a developmental disability caused by differences in the brain and has been associated with excessive inflammation, but the underlying mechanisms have not been fully explored. Mutations in SH3 and ankyrin repeat domains 3 (SHANK3) are associated with autism spectrum disorders. SHANK3 functions as a synaptic scaffolding protein. The gut and brain are linked by the vagus nerve. As described by Zhang et al., SHANK3 plays a vital role in maintaining body temperature following stress-induced changes. In addition, vagal SHANK3 can inhibit excessive inflammation following LPS challenge, providing new insights into inflammation dysregulation in some ASD patients. Further, they showed a novel interaction between SHANK3 and transient receptor potential melastatin 2 (TRPM2), an oxidant/stress sensor located in vagal sensory neurons. The authors presented results demonstrating that vagal sensory neurons may play an important role in SHANK3-dependent disorders.

Increased vascular permeability, infiltration of inflammatory cells, dysregulation of immune cell activation, a Ca2+ pannus, and unbearable pain hyperalgesia are common characteristics of rheumatoid arthritis (RA). The transient receptor potential channel (TRP) is a non-selective permeable cation channel that regulates the entry and intracellular Ca2+ signaling in multiple types of cells, including immune cells and neurons. Niu et al. present evidence of TRP involvement in RA pathogenesis and pain hyperalgesia by demonstrating that they act on vascular endothelial cells to induce joint swelling, neutrophil activation, and transendothelial migration, as well as bridging the reactive oxygen species (ROS)/TRPs/Ca2+/peptidyl arginine deiminases networks in accelerated citrullinated protein synthesis. The authors also showed that TRP subfamily expression in the nervous systems of joints affects inflammatory pain by influencing cold hyperalgesia and neuro-inflammation. The TRP pathway is an excellent target for providing analgesics and anti-inflammatory effects in RA.

The underlying mechanism of trigeminal neuralgia and subsequent anxiety are poorly understood, making it one of the most challenging forms of chronic pain to treat. Zhang et al. showed that tachykinin receptor 3 (NK3R) is involved in trigeminal neuralgia and pain-related anxiety in the lateral habenula (LHb), an endogenous NK3R ligand with high affinity. NK3R inhibition induced anxiety-like behavior and orofacial allodynia. When the infraorbital nerve is partially transected in a mouse model of trigeminal neuralgia (pT-ION), LHb neurons show hyperactivity. Allodynia and anxiety-like behaviors induced by pT-ION were attenuated by activating NK3R in the LHb. It was found that periaqueductal gray (fPAG) projects neurokinin B (NKB)-positive nerve fibers to the LHb. The results indicated that fPAG NKB projecting to LHb is involved in regulating orofacial allodynia and pain-induced anxiety behaviors. Inhibiting the release of NKB from the fPAG reversed the analgesic and anxiolytic effects of LHb. Trigeminal neuralgia may be relieved by targeting NK3R in this novel pathway.

Many malignancies can be treated with paclitaxel (PTX), including breast, ovarian, and lung cancers. Peripheral neuropathic pain (PINP) caused by PTX treatment is a serious side effect that limits its effectiveness in cancer patients. RvD1 (Resolvin D1), a specialized pro-resolving lipid mediator produced from docosahexaenoic acid by 15- and 5-lipoxygenase, is able to enhance macrophage phagocytosis, accelerate aging cell clearance, and increase macrophage production of IL-10. There is evidence that RvD1 and RvE1 can alleviate the pain caused by formalin in mice. As reported by Su et al., RvD1/N-formyl peptide receptor 2 (FPR2) up-regulates IL-10 in macrophages and IL-10 further activates Nrf2-HO1 pathway in neurons in the DRG, thereby reducing neuronal damage and PINP. It was found that systemic RvD1 supplementation could be a potential treatment strategy for PINP. Further research is required to establish whether RvD1 is responsible for neuropathic pain induced by other chemotherapy drugs, such as oxaliplatin and vincristine.

When macrophages are activated, itaconate is produced as a metabolite of the tricarboxylic acid (TCA) cycle. Besides being an effective immunomodulator, itaconate also has anti-inflammatory properties. Acod1 encodes the cis-aconitate decarboxylase immune response gene 1 (IRG1), which produces itaconate. Studies have shown that an itaconate derivative inhibits neuroinflammation and reduces chronic pain in mice. The team shows for the first time in Sun et al. report that endogenous itaconate increases and acts as an analgesic after nerve injury. Itaconate’s analgesic effect is dependent on the IL-10/endorphin pathway. It was found that pain hypersensitivity was more intense in chronic constriction injury (CCI) mice constructed with global *Irg1^-/-^* genes. The itaconate derivative 4-OI exhibits significant analgesic effects in male and female CCI mice by systemic or local application. This study used exogenous and endogenous itaconate to induce analgesia in a neuropathic pain model. In addition to promoting spinal cord IL-10 levels, 4-OI activates signal transducer and activator of transcription 3 (STAT3), thereby enhancing the expression of endorphins in the spinal cord. This pathway may mediate itaconate’s analgesic effect.

In summary, there is increasing evidence that nociceptor neuron-immune interactions play a crucial role in pain and inflammation. Their functional interactions may be necessary for preventing tissue damage and restoring homeostasis by recognizing damaging and/or harmful stimuli. In addition to peripheral injury sites, these neuroimmune interactions occur in the central nervous system. The sensory nervous system plays a crucial role in modulating host protective responses. Understanding how it interacts with immune cells may reveal new ways to treat and prevent diseases.

## Author contributions

P-HT wrote the manuscript and other authors edited the manuscript. All authors contributed to the article and approved the submitted version.

